# Reduction in stray radiation dose using a body‐shielding device during external radiation therapy

**DOI:** 10.1002/acm2.12035

**Published:** 2017-02-02

**Authors:** Shuxu Zhang, Shaohui Jiang, Quanbin Zhang, Shengqu Lin, Ruihao Wang, Xiang Zhou, Guoqian Zhang, Huaiyu Lei, Hui Yu

**Affiliations:** ^1^ Radiotherapy Center Affiliated Cancer Hospital & Institute of Guangzhou Medical University Guangzhou 510095 China

**Keywords:** body‐shielding device, IMRT, stray radiation dose, thermoluminescent dosimeter

## Abstract

With the purpose of reducing stray radiation dose (SRD) in out‐of‐field region (OFR) during radiotherapy with 6 MV intensity‐modulated radiation therapy (IMRT), a body‐shielding device (BSD) was prepared according to the measurements obtained in experimental testing. In experimental testing, optimal shielding conditions, such as 1 mm lead, 2 mm lead, and 1 mm lead plus 10 mm bolus, were investigated along the medial axis of a phantom using thermoluminescent dosimeters (TLDs). The SRDs at distances from field edge were then measured and analyzed for a clinical IMRT treatment plan for nasopharyngeal carcinoma before and after shielding using the BSD. In addition, SRDs in anterior, posterior, left and right directions of phantom were investigated with and without shielding, respectively. Also, the SRD at the bottom of treatment couch was measured. SRD decreased exponentially to a constant value with increasing distance from field edge. The shielding rate was 50%–80%; however, there were no significant differences in SRDs when shielded by 1 mm lead, 2 mm lead, or 1 mm lead plus 10 mm bolus (*P*>0.05). Importantly, the 10 mm bolus absorbed back‐scattering radiation due to the interaction between photons and lead. As a result, 1 mm lead plus 10 mm bolus was selected to prepare the BSD. After shielding with BSD, total SRDs in the OFR decreased to almost 50% of those without shielding when irradiated with IMRT beams. Due to the effects of treatment couch and gantry angle, SRDs at distances were not identical in anterior, posterior, left and right direction of phantom without BSD. As higher dose in anterior and lower dose in posterior, SRDs were substantial similarities after shielding. There was no significant difference in SRDs for left and right directions with or without shielding. Interestingly, SRDs in the four directions were similar after shielding. From these results, the BSD developed in this study may significantly reduce SRD in the OFR during radiotherapy, thus decreasing the risk of secondary cancers.

## 1 Introduction

In a linear accelerator, there are unintentional radiation sources, such as internal patient scatter, collimator scatter and leakage radiation.^1^ Therefore, when a target volume in cancer is exposed to prescription doses for a definitive or palliative goal, normal tissues and organs in patients are unavoidably exposed to stray radiation during photon‐beam radiotherapy with a linear accelerator, which increases the risk of inducing secondary cancers. The stray radiation dose (SRD) from photon‐beam radiotherapy has been studied both experimentally and theoretically for some time.^2^, ^3^, ^4^, ^5^, ^6^ Stray radiation, such as phantom scatter, collimator scatter, room scatter, leakage radiation, and even the therapeutic dose near the primary field, decreases exponentially with increasing distance, but does not disappear completely after attenuation in the patient's body. There is no effective way of making phantom scatter disappear or decrease. With new treatment modalities and further optimized delivery techniques, there is a serious and growing concern regarding radiation‐induced cancers and late tissue injury in cancer survivors, particularly in younger patients.^7^, ^8^, ^9^

Intensity‐modulated radiation therapy (IMRT) allows the dose to be concentrated in the tumor volume with a steep dose gradient while sparing normal tissues. However, the shortcoming of IMRT is that it increases the number of radiation‐induced secondary cancers at different levels.^10^, ^11^, ^12^, ^13^, ^14^ The risks of secondary cancers from stray radiation during IMRT have been evaluated by measuring the SRD outside the low‐energy X‐ray (<10 MV) IMRT field^15^ or the photoneutron dose from photonuclear reactions induced by high‐energy X‐ray radiation directly,^16^ or by MC simulations.^17^ Compared with conventional radiotherapy, IMRT may double the incidence of solid cancers in long‐term survivors.^13^, ^14^ The reason for this potential is that IMRT requires more monitor units (MU) than conventional treatments, to deliver the same amount of the prescription dose to the tumor. Due to a larger volume of normal tissue exposed to lower radiation doses in the OFR, such as collimator scatter and leakage radiation, the total‐body dose is larger. If an improvement in local tumor control is balanced with reduced acute toxicity, this outcome may be acceptable in older patients, but not in younger patients. Therefore, the main goal of radiation treatment is to identify an option which takes into account these two conflicting priorities, and not only reduces SRD to the surrounding normal tissue, but also focuses the prescription dose in the target volume.

Minimizing SRD in the OFR without compromising radiation treatment has received widespread attention with new treatment modalities, such as IMRT or image‐guided radiation therapy.^18^ Reducing the SRD in the OFR makes more sense for controlling radiation‐induced cancers. It is well known that low‐energy rays are easily shielded by high‐atomic‐number materials, such as lead and tungsten. Several groups have used lead as a shield to reduce SRD to the contralateral breast during tangential irradiation;^19^, ^20^ 55% and 65% of the relative dose can be reduced by 13 mm and 25 mm of lead shielding, respectively, when the contralateral breast is shielded. Unfortunately, lead shielding at these thicknesses is too heavy to use safely, and the lead shield does not reduce the SRD to normal tissues in the rest of the body. Researchers have proposed that thinner lead shielding is likely to have a significant protective effect on normal tissue.^19^, ^21^ Therefore, we developed a body‐shielding device (BSD) to reduce SRD in the OFR and reduce the risk of radiation‐induced cancers. SRD at distances from the field edge were measured and analyzed before and after shielding. In addition, SRD in the anterior, posterior, left and right positions of an anthropomorphic phantom were investigated with and without shielding, respectively. If the dose from phantom scatter can be reduced, then the BSD designed in this study may play an important role in reducing SRD.

## 2 Materials and methods

### 2.A Experimental apparatus

A 6 MV photon beam was produced by a linear accelerator (ONCOR Impression, Siemens, Germany). Dose measurements were performed using JR‐115B thermoluminescent dosimeters (TLDs; BNIF, Beijing, China), 3 × 3 × 0.8 mm^3^ in size, made of lithium fluoride (^6^Li) and doped with magnesium, copper, and phosphorus (LiF: Mg, Cu, P). The lower dose limit for the TLD was 10^−7^ Gy. The TLD system used in this study included a microprocessor‐controlled TLD reader (FJ347A, Beijing, China) and an annealing oven (FJ411A, Beijing, China). An adult anthropomorphic phantom (CFTLC, Chengdu, China) was used to simulate realistic stray radiation conditions during radiotherapy. The RW3 solid water‐slab phantom (IBA Dosimetry GmbH, Schwarzenbruck, Germany) with a physical density of 1.03 g/cm^3^ was also used in this study.

### 2.B TLD annealing and calibration

Prior to each irradiation, TLDs were annealed in an oven at 240 ± 2°C for 10 min, and then cooled rapidly to room temperature. The dosimeters were calibrated using a RW3 solid water‐slab phantom and the 6 MV X‐ray beam with a field size of 10 × 10 cm^2^ and a source‐skin distance of 100 cm. TLD output was measured using a FJ347A reader. The background signal was determined and subtracted from each TLD measurement. TLDs with a deviation in dose signal < ± 3% were used to measure the SRD in the OFR.

The dose signals of the chosen TLDs were recorded and averaged separately for different‐energy X‐ray beams (M_TLD_). The corresponding absolute doses applied to the TLDs under the 5 cm thick RW3 were measured by a plane‐parallel ionization chamber PCC40 (IBA Dosimetry GmbH) (M_5 cm_). The calibration factor for the TLDs for dose measurement was determined by the following formula: K = M_5 cm_/M_TLD._ If the relative sensitivity value of the dosimeter was greater than ± 10% of the mean value, it was rejected.

### 2.C Irradiation and measurement for experimental testing

During the experimental testing to choose the optimal shielding conditions, the solid water‐slab phantom was irradiated using 6 MV X‐ray beams with a gantry angle of 0°, field size of 10 × 10 cm^2^, 100 cm of source surface distance, dose rate of 300 MU/min and delivery dose of 100 MU. The SRD measurements were performed using TLDs along the medial axis and on the surface of the phantom, which were set at distances of 5, 10, 15, 20, 25, 30, 35, 40, 45, 50, 55, and 60 cm from the field edge. In addition, the measurements were obtained with and without shields, such as 1 mm lead, 2 mm lead, and 1 mm lead plus 10 mm bolus. To measure back‐scattering radiation due to the interaction between photons and lead, two groups of TLDs were placed on the surface of the 1 mm lead and 10 mm bolus, respectively. All measuring points were in a sagittal plane and were measured in triplicate.

### 2.D The production of BSD

The BSD was prepared using a 1 mm thick electrolytically purified lead sheet (99.994% pure). A layer of milky‐white paint was sprayed evenly on the surface of the lead to avoid lead contamination. When the paint was dry, the lead was pasted onto a 5 mm thick sheet of U‐shaped polymethyl methacrylate (PMMA) to form the bottom of the BSD. To reduce the back‐scattering of low‐energy stray radiation by lead, the lead was covered with a 10 mm thick tissue‐equivalent material (bolus) to form the top cover of the BSD.

**Figure 1:**
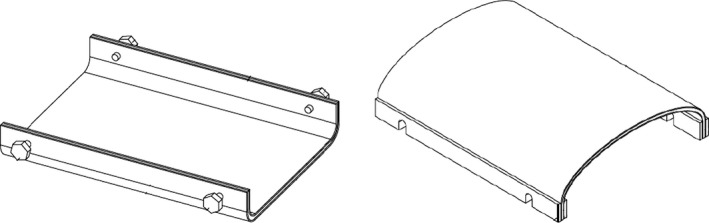
Illustration for body‐shielding device (BSD).

The side edges of the BSD were clamped by two layers of PMMA, and an arc supporting frame was fixed on the inner layer PMMA edge strip to support the weight of the top cover of the BSD and maintain its shape, as pure lead is soft and unable to support its own weight and that of the bolus. A set of locking nuts and corresponding interface ports were placed on the sides of the PMMA to lock the bottom and top cover and adjust the height of the top cover to fit the patient, to narrow the gap between the top cover and the patient's body surface. However, the shape of the BSD meant that it did not closely match the body surface, and a gap between the body and the top cover of BSD was inevitable, which could allow lateral‐scattering radiation to enter, thus exposing the patient to additional radiation. To reduce this lateral scattering, a medical rubber strip capsule—5 cm in diameter and filled with water—was fitted at the inner side of the BSD end port to seal the gap and absorb some of the scattering low‐energy rays. Illustration for BSD was displayed in Fig. 1.

### 2.E Irradiation and measurement for BSD

**Figure 2:**
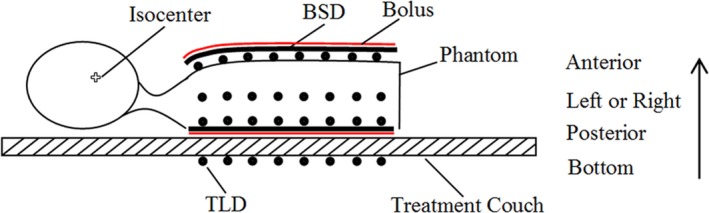
Experimental setup used to irradiate the TLDs with the 6 MV photon beam from the clinic linear accelerator.

The anthropomorphic phantom was fixed on a carbon fiber positioning plate using a thermoplastic mask, and then placed on the accelerator treatment couch. To investigate SRD in the OFR with or without shielding using the BSD, the phantom was irradiated with an IMRT clinical treatment plan for NPC. The prescription dose for isocenter irradiation was 200 cGy × 30 for 6 MV IMRT with 5, 7, and 9 radiation beams, respectively, which were designed for the same target in the same NPC patient. The details of treatment plans for NPC were shown in Table 1. These three IMRT plans provided 95% to 107% target coverage with acceptable organ at risk (OAR). The measurement points were set at distances of 5, 10, 15, 20, 30, and 40 cm from the field edge in the anterior, posterior, left, and right directions of the phantom, respectively (as shown in Fig. 2). In addition, a set of TLDs was placed at the bottom of the treatment couch with corresponding distances. The measurement points in the anterior, posterior, and bottom directions were in a sagittal plane, while a cross‐sectional plane was used for the measurement points in the right and left directions. All measurement points were measured in triplicate. The distance of 5 cm from the field edge corresponded to the clavicle.

**Table 1:** The details of treatment plans for NPC

Treatment plan	Number of beams	Gantry angles	Dose per fraction	Monitor units	Number of fractions
Siemens IMRT	5	0°, 72°, 144°, 216° and 288°	200 cGy	684 MU	30
Siemens IMRT	7	0°, 50°, 100°, 150°, 210°, 260° and 310°	200 cGy	669 MU	30
Siemens IMRT	9	0°, 40°, 80°, 120°, 160°, 200°, 240°, 280° and 320°	200 cGy	702 MU	30

### 2.F Statistical analysis

The statistical analysis of SRD was performed using SPSS 19.0 statistical software (SPSS Inc., Chicago, IL, USA). For each dose measurement, the mean response and standard error for the TLD group were calculated using OriginLab (OriginLab Corporation, Northampton, MA, USA). A t‐test was used to compare differences in anterior, posterior, left and right directions, and one‐way analysis of variance was used to analyze the changes in SRD as a function of distance and radiation beam. Differences were considered significant when *P*<0.05.

## 3 Results and discussion

### 3.A Experimental testing for optimal body‐shielding device (BSD)

**Figure 3:**
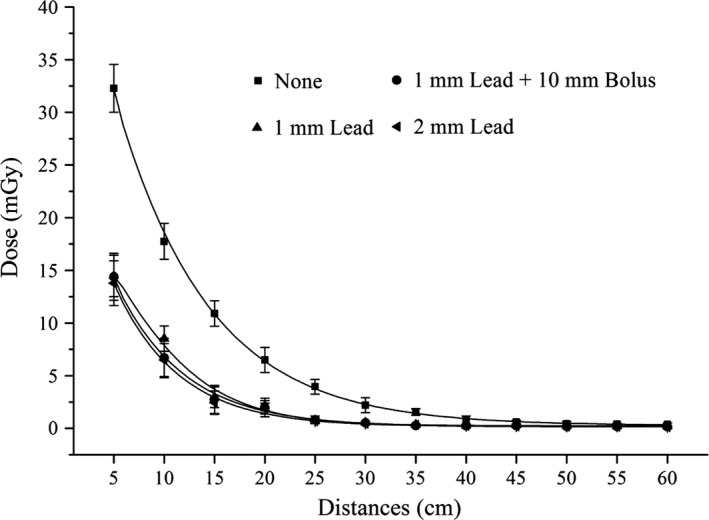
The distribution of SRD at distances from field edge when shielding or not with different shielding conditions.

The SRD profile along the medial axis from the field edge and in the 10 cm × 10 cm field is shown in Fig. 3. The curve fitting coefficients of the SRD data were all higher than 0.95. With increasing distance from the field edge, all SRDs decreased exponentially. These findings were consistent with those obtained by other researchers.^14^, ^22^ Shielding with 1 mm lead, 2 mm lead or 1 mm lead plus 10 mm bolus significantly reduced the SRD, and had a 50%–80% shielding rate. However, no significant differences in SRD were observed when shielding with 1 mm lead, 2 mm lead, or 1 mm lead plus 10 mm bolus were compared (*P*>0.05), suggesting that the use of 2 mm or thicker lead for shielding is unnecessary. For distances greater than 25 cm from the field edge, the SRD was constant after shielding. In addition, when shielding with lead, there was some back‐scattering of low‐energy stray radiation due to low‐energy X‐rays due to the interaction between photons and lead, as shown in Table 2 (*P*<0.05), which were absorbed by the 10 mm bolus placed on the surface of the lead (*P*>0.05). According to the optimal balance between tolerable weight and reduction in stray radiation, 1 mm lead plus 10 mm bolus was selected to prepare a BSD to reduce SRD in the OFR (as displayed in Fig. 1). As 6 MV photons are typically used in IMRT, it was not necessary to investigate the contribution of photoneutrons to total out‐of‐field dose, which are produced in the photonuclear interaction when a high‐energy radiation beam is used.^16^

**Table 2:** The SRD on the surface of 1 mm lead and 1 mm lead plus 10 mm bolus respectively

Distances (cm)	SRDs (mGy)		
	D_0_	D_1_	D_1‐B_
5	32.28 ± 2.27	41.64 ± 3.75	34.71 ± 3.13
10	17.75 ± 1.71	26.76 ± 2.69	18.12 ± 2.51
15	10.91±1.21	16.61 ± 1.88	11.10 ± 1.03
20	6.50 ± 1.19	10.16 ± 1.54	7.38 ± 1.93
25	3.95 ± 0.69	6.21 ± 0.89	4.44 ± 0.91
30	2.22 ± 0.71	3.45 ± 0.61	2.32 ± 0.77
35	1.57 ± 0.32	2.56 ± 0.55	1.63 ± 0.49
40	0.81 ± 0.37	1.30 ± 0.42	0.89 ± 0.52
45	0.60 ± 0.16	0.98 ± 0.39	0.59 ± 0.28
50	0.45 ± 0.20	0.71 ± 0.22	0.51 ± 0.37
55	0.43 ± 0.09	0.62 ± 0.18	0.44 ± 0.11
60	0.38 ± 0.13	0.58 ± 0.17	0.42 ± 0.09

D_0_ represents SRD without shielding; D_1_ represents SRD measured on surface of 1 mm lead; D_1‐B_ is for SRD measured on surface of 1 mm lead plus 10 mm bolus.

### 3.B SRD in the OFR irradiated with IMRT beams

An acceptable IMRT plan can usually be generated using 5 to 9 radiation beams. The optimal number of beams will depend on the complexity of the target shape and its proximity to critical structures.^23^ In our experiment, five, seven, and nine radiation beams were designed for the same target in the same NPC patient, which provided 95% to 107% target coverage with an acceptable spinal cord dose. When shielding with or without the BSD using the phantom, the changes in distance for SRD from different radiation beams were consistent with the results measured in the experimental testing, as shown in Table 3.

**Table 3:** SRD at distances from field edge when irradiated with different IMRT beams per fraction

Distances (cm)	SRDs in different IMRT beams (mGy)					
	Five beams		Seven beams		Nine beams	
	D_0–5_	D_1–5_	D_0–7_	D_1–7_	D_0–9_	D_1–9_
5	50.08 ± 19.57	28.37 ± 5.66	49.92 ± 21.49	25.09 ± 4.62	56.27 ± 14.53	28.59 ± 4.37
10	29.23 ± 8.28	17.02 ± 1.85	27.72 ± 10.77	17.79 ± 1.39	34.74 ± 9.63	18.05 ± 1.72
15	18.48 ± 5.01	11.68 ± 1.01	18.63 ± 7.50	9.49 ± 0.98	21.31 ± 4.31	11.34 ± 0.93
20	12.17 ± 3.51	6.96 ± 0.78	11.33 ± 4.21	6.45 ± 0.75	13.75 ± 3.69	7.72 ± 0.57
30	6.68 ± 1.78	3.72 ± 0.61	6.38 ± 2.05	3.52 ± 0.37	6.70 ± 1.31	3.93 ± 0.67
40	3.85 ± 0.83	2.15 ± 0.28	3.62 ± 0.84	2.15 ± 0.25	3.96 ± 0.75	2.40 ± 0.22

D represents SRD, while 0 and 1 represent before and after shielding, respectively, besides that 5, 7, and 9 are for the numbers of radiation beams, respectively.

When the different radiation beams were compared, there was no statistically significant differences in SRDs obtained with increasing distances from the field edge, respectively (*P*>0.05). The reason for this may be that the monitor units, 684 MU, 669 MU, and 702 MU applied per fraction in five, seven, and nine beams, respectively, were not substantially different and the prescription doses (200 cGy) to the target were the same in five, seven, and nine beams of IMRT.^24^ In addition, there were consistent reductions in these SRDs when the BSD was used. Although shielding rates at a distance of 30 cm and 40 cm were approximately 40%, the BSD reduced total SRDs in the OFR to nearly 50% of those without the BSD. This suggests that the BSD provided effective shielding, thus making patient treatment safer. Importantly, use of the BSD is also an easy way of decreasing SRD.

When the fraction of seven beams in a radiation treatment course for NPC is 30, total SRDs in the OFR would be 1.50 ± 0.64 Gy at a distance of 5 cm from the field edge when performed without shielding, and up to 2.51 Gy at certain measurement points. According to the dose–response relationship for radiation‐induced cancers, these SRDs are in the linear region where a dose on solid tumor mortality is consistent with linearity up to about 2.5 Gy with a risk of approximately 10%/Gy.^25^ Although the dose decreases exponentially at a range of distances and then reaches a constant value, total SRDs in the OFR may also reach 0.51 Gy at a distance of 20 cm from the field edge when 30 fractions of treatment are administered without shielding. It is thought that there is no dose threshold for radiation‐induced cancers. Although SRD is lower at distances over 20 cm from the field edge, corresponding to the chest and abdomen, it is still necessary to reduce the dose as much as possible. For example, the risk of secondary cancers in 6000 cases of cervical cancer treated with radiotherapy was higher than that in the control group without radiotherapy.^26^ Based on 348 cases of secondary stomach cancer, the excess relative risk was approximately 0.54/Gy after 5 years in patients with cervical cancer treated with radiotherapy.^27^

After shielding with the BSD in all 30 fractions of seven beams, total SRDs at 5 cm from the field edge was 0.75 ± 0.14 Gy, and was about 0.19 Gy at a distance of 20 cm from the field edge, which were much lower than those without the BSD. The low‐energy photons and electrons in the OFR, and some high‐energy scatter radiation, can be easily absorbed using the BSD. Thus, the BSD can achieve an effective reduction in unwanted dose. Based on the linear dose–response relationship at doses ranging from 0.1 Gy to 2.5 Gy, the risk of secondary cancers can be reduced due to dose reduction using the BSD.

### 3.C SRD in the anterior, posterior, left and right direction of phantom

**Figure 4:**
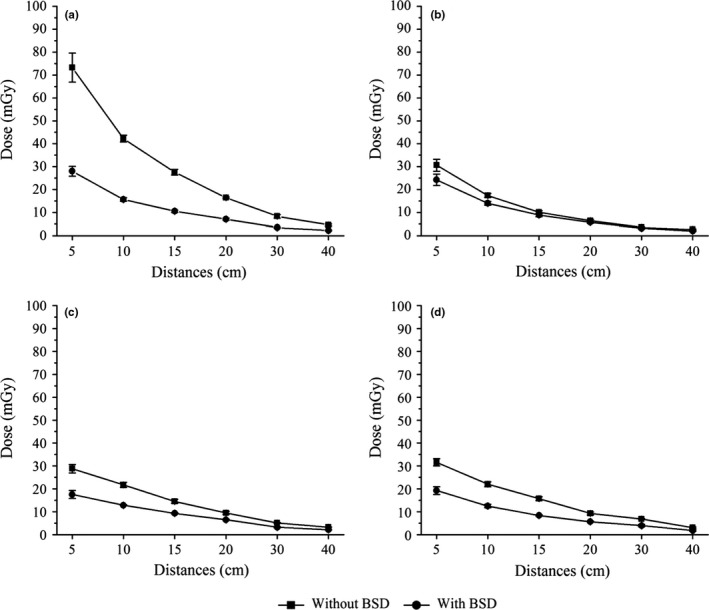
SRDs in anterior, posterior, left and right of phantom when shield or not, A, B, C, and D were for anterior, posterior, right, and left, respectively.

During radiation treatment, stray radiation in the OFR, such as phantom scatter and collimator scatter, results in scattering internally in various directions. As X‐rays are emitted from various gantry angles during per fraction of treatment, radiation leaks in various directions. The spatial distribution of stray radiation varies with changes in distance and gantry angle. Measured with or without the BSD and from seven radiation beams with gantry angles of 0°, 50°, 100°, 150°, 210°, 260°, and 310°, SRDs at distances from the field edge were not identical in the four directions measured, namely anterior, posterior, left, and right (as shown in Fig. 4).

In the anterior direction, SRDs without shielding at distances were higher than those in the posterior direction, respectively (*P*<0.05). However, the shielding rate of the BSD was 50%–60% and 10%–20% for the anterior and posterior direction, respectively. In addition, SRDs without shielding in the posterior direction were lower than those at both sides (left and right), without a significant difference at a distance of 5 cm due to the effect of the therapeutic dose from near the primary field. This may be attributed to the effects of the linac treatment couch which was made of carbon fiber.

From Table 4, it can be seen that SRDs without shielding at distances in the posterior direction were significantly lower than those at the bottom of the treatment couch (*P*<0.05). TLDs were placed in the same sagittal plane of the phantom with corresponding distances from the field edge to each other. If the linac treatment couch had no effect, there would be no significant difference between the dose in the posterior direction and the bottom of the treatment couch. It is well known that carbon fiber has a high absorption coefficient for low‐energy rays. The SRDs at distances in the anterior and posterior directions, which were measured under BSD shielding, showed significant similarities, respectively (*P*>0.05). These findings show that low‐energy stray radiation in the OFR can easily be absorbed by the BSD and carbon fiber couch. However, the linac treatment couch did not cause significant attenuation of 6 MV energy rays during radiation treatment, as only about 2% was absorbed.

**Table 4:** The SRD in OFR for posterior direction of phantom and bottom of treatment couch, respectively

Distances (cm)	Out‐of‐field doses (mGy)	
	D_B_	D_P_
5	57.39 ± 2.24	30.61 ± 2.64
10	30.70 ± 2.48	17.43 ± 0.91
15	18.36 ± 1.44	10.13 ± 0.64
20	11.50 ± 0.56	6.48 ± 0.46
30	5.96 ± 0.37	3.60 ± 0.19
40	3.74 ± 0.56	2.72 ± 0.28

D_B_ represents SRD measured at bottom of treatment couch, while D_P_ is for SRD measured in posterior direction.

In addition, SRDs at distances in the left and right direction showed significant similarities with and without shielding, respectively. The reason for this may be that the gantry angle was uniformly distributed on both sides, 50°, 100°, and 150° for the right and 210°, 260°, and 310° for the left, leading to insignificant spatial distribution of stray radiation on both sides. However, SRDs at distances in the left and right direction were lower than those in the anterior direction before shielding, respectively (*P*<0.05). This may be attributed to the impact of the carbon fiber couch and gantry angles. When the SRDs at 0° and 180° were compared, the SRD decreased as the gantry angle reached 90° and 270°.^28^ Furthermore, a portion of low‐energy stray radiation in the OFR was absorbed by the carbon fiber couch when the radiation beams were 100°, 150°, 210°, and 260°. The differences in the phantom profile in the anterior direction and both sides cannot be ignored. Interestingly, SRDs in the four directions were similar without statistical significance after BSD shielding when distances from the field edge exceeded 10 cm, except the SRD at a distance of 5 cm, which may have been affected by the therapeutic dose due to the proximity of the primary field. These findings showed that the BSD played an effective role in stray radiation shielding, particularly in the anterior direction. Although the BSD was effective in the anthropomorphic phantom, further studies are needed to determine the shielding effects of the BSD in patients.

## 4 Conclusion

A reduction in unwanted stray radiation in the OFR is in accordance with keeping radiation exposure as low as reasonably achievable. The BSD developed in this study may significantly reduce stray radiation in the OFR, particularly in the anterior direction, and thus decrease the risk of radiation‐induced cancers. The BSD requires further improvement, and further clinical study is pending to demonstrate its performance.

## Competing interests

The authors declare that they have no competing interests.

## Authors' contributions

S.Z. was responsible for the primary concept and the design of the study; S.Z., S.J., Q.Z., X.Z., R.W., G.Z., H.L., H.Y., and S.L. performed the data capture and analysis. S.Z. and Q.Z. drafted the manuscript; all authors revised the manuscript. All authors have read and approved the final manuscript.
